# Time from HIV diagnosis to commencement of antiretroviral therapy as an indicator to supplement the HIV cascade: Dramatic fall from 2011 to 2015

**DOI:** 10.1371/journal.pone.0177634

**Published:** 2017-05-16

**Authors:** Nicholas A. Medland, Eric P. F. Chow, James H. McMahon, Julian H. Elliott, Jennifer F. Hoy, Christopher K. Fairley

**Affiliations:** 1 Melbourne Sexual Health Centre, Central Clinical School, Monash University, Melbourne, Australia; 2 Alfred Hospital, Central Clinical School, Monash University, Melbourne, Australia; National and Kapodistrian University of Athens, GREECE

## Abstract

**Introduction:**

The HIV care cascade is increasingly used to evaluate HIV treatment programs at the population level. However, the cascade indicators lack the ability to show changes over time, which reduces their utility to guide health policy. Alternatives have been proposed but are complex or result in a delay in results. We propose a new indicator of ART uptake, the time from HIV diagnosis to commencement of ART, and compare it to the existing cascade indicator of proportion of patients on treatment and the WHO proposed cohort cascade indicator of proportion of patients on treatment within one year of diagnosis.

**Methods and materials:**

Records from patients from the two largest HIV treatment centres in the state of Victoria, Australia (Melbourne Sexual Health Centre and The Alfred Hospital Department of Infectious Diseases) from 2011 to 2015 were extracted. The intervals between date of diagnosis, entry into care and initiation of ART were compared.

**Results and discussion:**

From 2011 to 2015 the proportion of in-care patients who were on ART rose from 87% to 93% (p<0.0001). From 2011 to 2014, the proportion of patients in care and on ART within one year of diagnosis increased from 43.4% to 78.9% (p = 0.001). The median time from diagnosis to ART fell from 418 days (IQR: 91–1176) to 77 days (IQR: 39–290)(p<0.001) by calendar year in which ART was commenced.

**Conclusions:**

From 2011 to 2015 there were substantial and clinically important falls in the median time from diagnosis to commencing ART in those that commenced ART. The size of this dramatic change was not apparent when only reporting the proportion of patients on ART. Time to ART is a useful indicator and can be used to supplement existing cascade indicators in measuring progress toward universal ART coverage.

## Introduction

Antiretroviral therapy (ART) has been shown to benefit all individuals living with HIV, including those with asymptomatic infection and higher CD4 cell counts [1, 2]. Furthermore, ART has been demonstrated to reduce HIV transmission in clinical trials of serodiscordant partners [3–5]. Ecological studies have also described reduced HIV incidence in the setting of increased population ART coverage [6]. The importance of the individual health benefits of ART and treatment as prevention have have led to the development of the cascade of HIV care, which is increasingly used to represent and investigate what proportion of the HIV-infected population are diagnosed, linked and retained in care, receiving ART and virologically suppressed [7]. These cascades can be used to identify where interventions to improve coverage of clinical care should occur and measure how successful they are. National and international programs are now looking to the cascade to guide and measure interventions to achieve high ART coverage [8, 9].

However, the HIV care cascade is not without limitations. Firstly, it is a cross-sectional representation of all patients who have ever been diagnosed with HIV. Trends in early diagnosis, linkage to care and initiation of ART in recently diagnosed individuals may be difficult to distinguish because of the relatively larger numbers of patients on stable suppressive therapy, all equally represented in the cross-sectional cascade.

Secondly, the HIV cascade uses aggregate data and cannot account for the dynamics of the populations being represented. Aggregate data cannot tell how long individuals have spent in each step of the cascade before moving to the next step. When considering the preventive effect of HIV treatment, factors which directly affect the duration of infectiousness (the period between infection and treatment) are particularly relevant.

Thirdly, when complete population-based data is not available, as is the case in most jurisdictions, apparent changes in the cascade over time are difficult to interpret. In fact, national or jurisdictional cascade data may not be able to determine trends in antiretroviral uptake. For example, the Australian cascade estimates that 73% of 23,800 individuals living with diagnosed HIV in 2015 were receiving ART in 2015 *versus* 77% of 23,100 individuals in 2014 [10, 11]. However, the limitations of the data sources and changes in methodology do not allow any inferences to be drawn from the apparent fall from 77% in 2014 to 73% in 2015 [12].

Other ways of measuring treatment coverage may be required if the progress toward treatment as prevention goals is to be meaningfully charted. The World Health Organisation (WHO) has proposed a cohort-based HIV cascade focussing on treatment uptake in individuals diagnosed with HIV during a specified year [13]. However, even a yearly cohort cascade will be relatively insensitive to increased ART uptake in patients initiating ART earlier than one year after diagnosis. In addition to the number or proportion of patients who are diagnosed, in care and on ART, the movement of individuals over time from infection through diagnosis to care and ART could provide information about the effectiveness of services in reducing the duration of infectiveness in individuals. Also, most clinic populations contain a diverse group of patients who are not yet receiving ART, including the recently diagnosed, those yet to move toward early treatment and patients who decide against early treatment for whatever reason.

Studies specifically designed to examine changes in time to ART initiation over time have been conducted[14, 15]. These are illuminating investigations of the dynamics of the HIV care cascade. However, they are statistically complex and hence less useful to compare clinic level progression toward universal ART coverage. Information on ART uptake will be relevant at the jurisdictional level to guide public health policy and evaluation of interventions. However, clinics and services will also need indicators which are readily and rapidly available to benchmark their performance and their contribution to larger public health goals.

The ideal indicator would be sensitive to time changes, produce results quickly at the end of a period of interest and be able to account for both recently diagnosed and existing untreated patients. The indicator should be easy to collate at both the local clinic level and at the jurisdictional level. We propose a new indicator of ART uptake, the time from HIV diagnosis to commencement of ART, which meets these criteria and can meaningfully supplement the current HIV treatment cascade at both a jurisdictional and service or clinic level.

The aim of this study was to compare antiretroviral coverage and uptake using different methods: the HIV care cascade indicator of proportion of patients on ART, the WHO proposed cohort cascade method of proportion of patients on ART within one year of diagnosis and the newly proposed indicator of time to commencing ART.

## Methods and materials

Alfred Health provides two HIV outpatient services: Melbourne Sexual Health Centre (MSHC) and the Department of Infectious Diseases (DID), located in separate campuses in Melbourne, a city of four million people in the state of Victoria, Australia. The MSHC has a specialist HIV treatment clinic embedded within a public sexual health service, which also performs high volume HIV and sexual health testing services. The DID provides an HIV outpatient service inside a large public tertiary care hospital. Combined, these two centres manage the care of approximately 2450 patients, of an estimated 6300 people living with HIV in Victoria [16].

Each of these sites has an electronic data system with up-to-date patient treatment information. 2011 to 2015 was selected as the study period because MSHC has used an electronic health record since 2011. We extracted data from records of patients with a recorded date of first ART any time between 1 January 2011 and 31 December 2015. The following data were recorded for each patient: the date of HIV diagnosis, the date of their first visit for specialist HIV medical care, the date of commencement of ART, the lowest ever CD4 count, age at the time of commencement of ART, gender, country of birth, and self report HIV exposure risk. We excluded patients already on ART who transferred their care, because the date of care could not be determined.

Patients were excluded if the diagnosis date or ART commencement date was not recorded and was unable to be determined from the record, if the recorded ART date was inconsistent with recorded viral load data or other data inconsistencies were unable to be resolved. For patients at the DID, these fields are collected prospectively by a dedicated data manager from clinical information provided by the treating physician or clinical nurse. Data are checked at the time of entry into the dedicated database. MSHC uses a customised clinical practice management system into which these fields are entered by the treating physician or clinical nurse at the time of the patient contact. At MSHC, this data was checked manually for accuracy at the time of extraction by referring to the original clinical notes, results and records.

For the HIV care cascade indicator, we calculated the proportion of all in-care patients who were on ART. For each year from 2011 to 2015, we included all patients with at least one visit during that year and determined if, on December 31^st^ of that year, they had received or not yet received ART.

For the HIV cohort cascade, we calculated the proportion of all in-care patients who were on ART within one year of diagnosis. For each year 2011 to 2014, we examined all patients in care who were diagnosed within that year and determined if they had commenced ART within one year of that date.

For the time-based indicators, we calculated time from diagnosis to ART as the number of days between the date of diagnosis of HIV infection to the first day of ART. Records of patients receiving care at both sites or transferring between sites were merged into the record of the centre they were attending at first day of ART. We calculated the time from diagnosis to care as the number of days between the date of diagnosis of HIV infection to the date of the first visit at either specialist centre and the time from care to ART as the time in days between the date of the first visit at either specialist centre to the first date of ART.

We analysed these indicators according to year of diagnosis and year of commencing ART. The latter would include patients diagnosed before the study period and monitored until commencing ART during the study period. We determined the median time from diagnosis to ART, from diagnosis to care and care to ART. Statistical significance was determined using the Jonckheere-Terpstra Test, and the SPSS statistical software package, to compare the trend in yearly median values.

We combined the data from two different types of treatment centre so as to increase the generalizability of findings. In representing the data, the sites are referred to as Centre A and Centre B, as the study was not designed to make comparisons between the two centres which have a different structure as well as patient group.

This study was approved by the Alfred Hospital Human Ethics Committee (approval number 375/15). Ethics approval included waiver of individual patient consent for the collection and aggregate reporting of retrospective data.

## Results and discussion

729 patients commenced ART between 1 January 2011 and 31 December 2015 after excluding 12 patients whose date of diagnosis could not be determined and 16 patients whose date of commencement of ART could not be determined. 11 patients received care at both sites and were included at the site where the ART was first prescribed (3 to Centre A and 8 to Centre B). Of these 729 patients, 512 had also been diagnosed with HIV between 2011 and 2015 and 217 had been diagnosed before 2011.

Patients starting treatment at Centre A were younger, less likely to be born in Australia and had a higher CD4 nadir than patients starting treatment at Centre B Table 1.

**Table 1:** Characteristics of patients commencing ART from 2011 to 2015.

	All (N = 729)	Centre A (N = 470)	Centre B (N = 259)	p-value
Age, mean (±SD)	35.8 (±10.4)	33.8 (±9.5)	39.3(±10.8)	<0.001^
Gender, n (%)^#^				
* Male*	680 (93.3%)	440 (93.6%)	240 (92.7%)	0.623^#^
* Female*	49 (6.7%)	30 (6.4%)	19 (7.5%)	
MSM, n (%)				
* Yes*	521 (83.5%)	365 (85.9%)	156 (78.4%)	0.19^#^
* No*	103 (16.5%)	60 (14.1%)	43 (21.5%)	
Country of birth, n(%)				
* Australia*	393 (57.2%)	229 (50.8%)	164 (69.5%)	<0.001^#^
* Overseas*	294 (42.8%)	222 (49.2%)	72 (30.5%)	
Nadir CD4 cell count/uL, mean (±SD)	349 (±206)	372 (±215)	306 (±182)	<0.001^

^Two-sample t test was used to compare the mean between two groups.

^#^ Pearson’s chi-square test was used to compare proportions between two groups.

### Proportion of patients on ART

From 2011 to 2015, the proportion of patients in care and on ART increased from 86.6% of 1779 to 93.4% of 2295 patients (p<0.001) Table 2.

**Table 2:** Proportion of in-care patients on ART at 31 December of each year.

	2011	2012	2013	2014	2015	
Total patients in care*	1779	1943	2118	2206	2295	
Number of patients on ART	1541	1708	1839	2015	2144	
Proportion of patients on ART	86.6%	87.9%	86.8%	91.3%	93.4%	<0.0001^#^

*Total number of patients in care on December 31 of that year.

^#^ Cochrane-Armitage trend test.

### Proportion of patients on ART within one year of diagnosis

From 2011 to 2014, the proportion of patient in care and on ART within one year of diagnosis increased from 43.4% to 78.9% (p<0.001) Table 3.

**Table 3:** Proportion of in-care patients on ART within one year of HIV diagnosis.

	Total	2011	2012	2013	2014	
Patients diagnosed*	543	145	146	119	133	
Patients on ART within one year	329	63	77	84	105	
Proportion of patients on ART within one year	60.6%	43.4%	52.7%	70.6%	78.9%	0.001^#^

^#^ Cochrane-Armitage trend test.

*includes patients who did not receive treatment

### Time from diagnosis to ART

From 2011 to 2015, the median time from diagnosis to ART fell significantly from 418 days (IQR: 91–1176) to 77 days (IQR: 39–290) in 729 patients commencing treatment over this time. The median time from diagnosis to care fell from 34 days (IQR: 9–346) to 14 days (IQR: 5–29) (p<0.001) and the median time from care to ART fell from 140 days (IQR: 36–610) to 51 days (IQR: 21–216) Table 4.

**Table 4:** Time from diagnosis to treatment, from diagnosis to care, and care to treatment, age and CD4 nadir in patients commencing ART from 2011 to 2015.

Year of ART* number	2011–2015 n = 729	2011 n = 125	2012 n = 147	2013 n = 142	2014 n = 168	2015 n = 147	p-value
Days from diagnosis to ART, median [IQR]	220 [56–883]	418 [91–1176]	430 [116–1337]	222 [63–618]	194 [48–997]	77 [39–290]	<0.001^
Days from diagnosis to care, median [IQR]	21 [8–125]	34 [9–346]	33 [11–284]	19 [9–120]	21 [7–104]	14 [5–29]	<0.001^
Days from care to ART, median [IQR]	92 [30–454]	140 [36–610]	141 [39–602]	105 [37–519]	61 [25–360]	51 [21–216]	<0.001^
Age, mean (±SD)	35.7 (±10.4)	37.8 (±10.9)	37.2 (±10.4)	36.1 (±10.8)	35.5 (±9.8)	32.6 (±9.3)	<0.001^#^
Nadir CD4 cell count/uL, mean (±SD)	340 (±188)	274 (±166)	308 (±138)	292 (±146)	362 (±199)	439 (±220)	<0.001^#^

Notes

^ Statistical significance of trend was determined using the Jonckheere-Terpstra Test.

# Statistical significance of trend was determined by one way analysis of variance (ANOVA).

*All patients with a reported date for first ART between 01 JAN 2011 and 31 DEC 2015.

The same trends in time to commencing ART were observed in both centres over all time periods except for patients starting treatment with lower CD4 counts in Centre B. The median time to ART for patients diagnosed in 2011 was 301 days (IQR:88–629). This fell to 56 days (IQR: 36–147) [36–147] for patients diagnosed in 2014 and who had started treatment before the end of the study period, 31 December 2015 (p<0.001).

**Fig 1:**
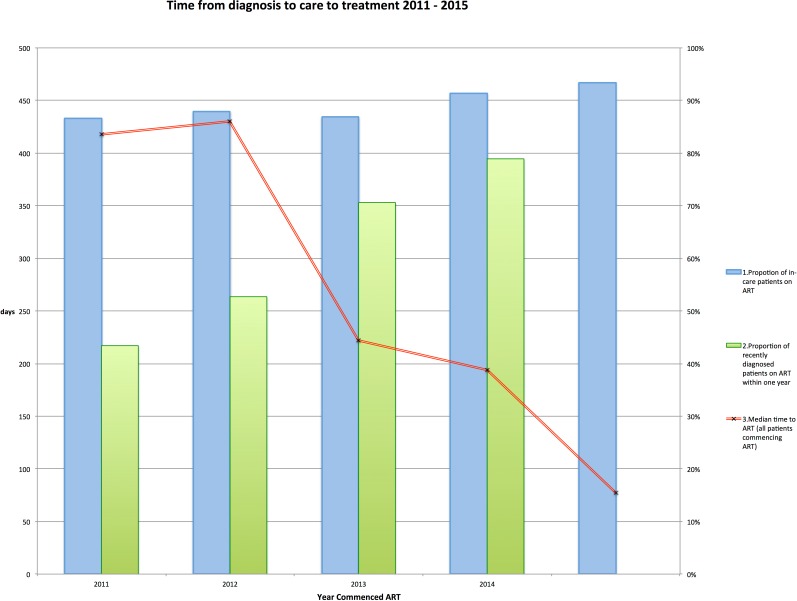
Proportion of patients on ART and time to ART 2011 to 2015. 1.Proportion of in-care patients on ART: percentage of all patients in care receiving ART on Dec 31 of that year. 2.Proportion of recently diagnosed patients on ART within one year: Proportion of patients diagnosed with ART between 01 Jan and 31 Dec of that year who have commenced ART within one year of diagnosis. 3.Median time to ART (all patients starting ART in that year): time from diagnosis to ART for all patients commencing ART between 01 Jan and 31 Dec.

The three indicators (proportion of in care patients on ART, proportion of newly diagnosed patients on ART within 1 year of diagnosis and time from diagnosis to ART) are shown together in Fig 1.

Our data demonstrated substantial and clinically important falls in the median time from diagnosis to ART from more than one year to less than three months for patients starting treatment from 2011 to 2015 and from 10 months to 1.5 months in patients diagnosed from 2011 to 2015. From a public health perspective this equates to a considerable reduction in the time newly diagnosed individuals had a detectable viral load and were infectious. This dramatic change was largely unapparent on the cascade indicator, the proportion of patients taking ART, which increased from 87% to 93% over the same period although was more obvious in the cohort cascade Fig 1. The implications of these findings are that centres should consider using time to ART, rather than just the proportion of patients on treatment, to benchmark their performance against other centres and establish indicators of best practice.

The time from diagnosis to care fell from more than five weeks to approximately two weeks from 2011 to 2015. This includes the period between the patient presenting for testing and receiving a confirmed positive result and the waiting time for an appointment at the specialist centre. Although this time could be shortened by reducing the waiting time for results and specialist appointments, further reductions will likely only have a small overall impact on the total time from diagnosis to ART as compared to the dramatic reductions in time from diagnosis to ART commencement already observed.

Several factors will have caused this dramatic decline in time to ART. Firstly, awareness of the effect of ART on transmission has steadily grown since the release of the HPTN 052 study in 2011 and other subsequent studies [3–6]. Secondly, international and local treatment recommendations changed over this period. From March 2012, the United States Department of Health and Human Services (DHSS) guideline for the use of ART recommended ART for all people with HIV, although with only moderate supportive evidence (BIII, or a moderate strength recommendation based on expert opinion). In July 2015, the Strategic Timing of Antiretroviral Treatment (START) study and the TEMPRANO study released data supporting immediate versus delayed treatment initiation [1, 2]. Soon after, the United States DHSS guidelines strengthened their recommendation to the highest degree of supportive evidence (A1 or multiple randomised controlled trials) [17]. The Australasian Society of HIV Medicine (ASHM), the peak body supporting the HIV clinical care provider workforce, offers a commentary on the DHSS guidelines and rapidly incorporated the latest change in 2015 [18]. Surveys of Australian physicians treating HIV demonstrated a rise an increase in acceptance of early ART initiation[19]. Thirdly, funding restrictions for ART in Australia were relaxed over the same period. In Australia, ART is subsidized through the national Pharmaceutical Benefits Scheme. Prior to 2014, prescribing of ART was restricted to patients with a CD4 cell count less than 500 or a viral load greater than 10,000 copies. The viral load requirement was dropped in May 2013 and the CD4 threshold removed in May 2014 [20].

There was a dramatic and continuous change in time to ART from 2011 to 2015 with significant implications for treatment as prevention at a community level. The cascade indicator, proportion of patients on treatment, was the least sensitive to this change. The proportion of all patients on ART is driven by several factors, including the number of patients already on ART. The cascade includes cumulative, aggregated and cross-sectional data from all patients, irrespective of when they were diagnosed or when they commenced treatment. Therefore, significant changes in the ways that newly diagnosed patients start treatment may be less visible when those patient numbers are combined with the relatively larger numbers of patients already on treatment.

To address this deficiency, the WHO also recommends a cohort-based HIV cascade, which is based on those diagnosed living with HIV and diagnosed in a given year [13]. In our analysis, the cohort cascade indicators did indeed show a larger change than the cross-sectional cascade. However, these data cannot be collected until a year has elapsed from the end of the time period in question. Also, future reductions in median time to ART can be expected to have lesser effects on the proportion on ART after 1 year. For examine, the median time to ART for patients diagnosed in 2014 was 56 days (IQR: 36–147) and by 2015 it had fallen to 46 days by 2015 (IQR:31–75). This further reduction could only be expected to produce a minor increase the proportion on ART within one year.

We were interested in the time to ART as an indicator of ART uptake. Time from diagnosis to virological suppression by calendar year of diagnosis at the jurisdictional level has recently been published [14]. This indicator also is highly sensitive to change in ART uptake. However, mean or median time to ART in this indicator is biased toward shorter time to treatment in more recent year of diagnosis, because patients very recently diagnosed have had less opportunity to commence ART. To circumvent this limitation, analysis must be deferred until at least one year after the final date of diagnosis and Kaplan-Meier time survival analyses deployed. Time to ART by year of diagnosis is a useful indicator for more in depth analalyses as the jurisdictional or the academic level but the delay in results and the complex statistics required means that it is unlikely to be useful at the clinic level.

We studied the time to ART by the calendar year in which patients started ART. Should rates of ART coverage approach 100%, almost all patients commencing ART will be newly diagnosed. However, in most centres previously diagnosed and untreated patients represent a sizeable proportion of the untreated population. This indicator accounts for a mix between newly diagnosed and previously diagnosed patients, and hence is closer to the experience of everyday clinical practice. It has the additional benefits of being available for calculation immediately at completion of the study period.

This indicator may also be more feasible to implement in resource limited settings or in settings with limited data systems, as it does not involves the creation of a retrospective cohort.

Each of these indicators has the benefit of being highly sensitive to changes in clinical practice, producing a result which is meaningful to the treatment as prevention and applicable to individual patients care. Ensuring individual patients rapidly entering care and then commence ART as soon as they are fully ready is important to increasing overall ART coverage. This study makes the case for directly measuring these intervals in addition to aggregating patients in the HIV care cascade.

Our findings are subject to certain limitations. Firstly, there were some differences between patients from the two centres most notably the lower CD4 count in patients starting ART between 2011 and 2015 in Centre B. This centre, because it is associated with an inpatient unit and general hospital consultation service, services a population with more advanced disease, psychosocial complexity and medical comorbidity. These patients, in whom deferral of ART under then current guidelines was advisable and in the best interest of the patients, will appear in the data as patients with a lower CD4 nadir and a longer time to ART. Secondly, we reported median treatment times to accommodate the highly skewed data, with small numbers of individuals with longer time from diagnosis to treatment. Thirdly, patients in Australia seek treatment in a range of different settings. We only examined patient care at the two largest treatment centres in this state, both of which are publicly funded. Many patients also receive treatment in private primary care clinics. Finally, the time from diagnosis was only calculated in patients who commenced ART and was not able to account for patients who have never entered specialist care or have never commenced antiretroviral therapy. However the proportion of diagnosed clients on treatment (93%) in 2015 was high suggesting that this latter bias was minimal.

## Conclusion

Time from HIV diagnosis to ART, from diagnosis to care, and from care to ART have fallen substantially and significantly. Time to ART as an indicator is sensitive to changes in treatment patterns, and readily and immediately available. As cascades may not reflect these changes, we recommend that centres use these metrics to benchmark their performance against other centres and establish indicators of best practice.
